# IDE Degrades Nociceptin/Orphanin FQ through an Insulin Regulated Mechanism

**DOI:** 10.3390/ijms20184447

**Published:** 2019-09-10

**Authors:** Gabriele Antonio Zingale, Francesco Bellia, Ikhlas Mohamed Mohamud Ahmed, Przemyslaw Mielczarek, Jerzy Silberring, Giuseppe Grasso

**Affiliations:** 1 Department of Chemical Sciences, University of Catania, 95125 Catania, Italy; 2 Institute of Crystallography, National Research Council, 95126 Catania, Italy; 3 Maj Institute of Pharmacology, Polish Academy of Sciences, Smetna 12, 31-343 Krakow, Poland; 4 Department of Biochemistry and Neurobiology, AGH University of Science and Technology, Mickiewicza Ave. 30, 30-059 Krakow, Poland; 5 Centre for Polymer and Carbon Materials, Polish Academy of Sciences, M.Curie-Sklodowskiej 34, 41-819 Zabrze, Poland

**Keywords:** Insulin-degrading enzyme, nociceptin, pain threshold, diabetes, proteolytic enzyme, pain transmission, rat spinal cord, cysteine protease, metalloprotease, neuropeptide

## Abstract

Insulin-degrading enzyme (IDE) was applied to catalyze hydrolysis of Nociceptin/Orphanin 1-16 (OFQ/N) to show the involvement of the enzyme in degradation of neuropeptides engaged in pain transmission. Moreover, IDE degradative action towards insulin (Ins) was inhibited by the OFQ/N fragments, suggesting a possible regulatory mechanism in the central nervous system. It has been found that OFQ/N and Ins affect each other degradation by IDE, although in a different manner. Indeed, while the digestion of OFQ/N is significantly affected by the presence of Ins, the kinetic profile of the Ins hydrolysis is not affected by the presence of OFQ/N. However, the main hydrolytic fragments of OFQ/N produced by IDE exert inhibitory activity towards the IDE-mediated Ins degradation. Here, we present the results indicating that, besides Ins, IDE cleaves neuropeptides and their released fragments act as inhibitors of IDE activity toward Ins. Having in mind that IDE is present in the brain, which also contains Ins receptors, it cannot be excluded that this enzyme indirectly participates in neural communication of pain signals and that neuropeptides involved in pain transmission may contribute to the regulation of IDE activity. Finally, preliminary results on the metabolism of OFQ/N, carried out in the rat spinal cord homogenate in the presence of various inhibitors specific for different classes of proteases, show that OFQ/N proteolysis in rat spinal cord could be due, besides IDE, also to a cysteine protease not yet identified.

## 1. Introduction

Diabetes mellitus (DM) is a disease that is spread all around the world and represents a global public health problem [1]. Two major diabetes types can develop (not considering gestational diabetes): type 1 and type 2, characterized by a lack of insulin (Ins) production and by Ins resistance, respectively. Common symptoms and problems of untreated diabetes are excessive thirst and hunger, visual disturbances, frequent urination (from urinary tract infections or kidney problems), weight loss or gain, fatigue, risk of heart disease and infections, irritability, slow-healing wounds, damaged blood vessels, and nerve damage [2,3,4]. Moreover, diabetic patients have a high pain threshold [5,6], whereas Ins has an antinociceptive effect, widely reported in the literature [7].

Nociceptin/Orphanin FN(1-17) (OFQ/N; FN denotes the presence of phenylalanine (F) at the N-terminal position and glutamine (N) at the C-terminal side) is considered to be a new member of the opioid family, showing sequence homology with the main endogenous opioid peptides of mammals, especially with dynorphin A. The primary amino acid sequences of OFQ/N and certain other endogenous peptides exhibit a common characteristic: the N-terminal tetrapeptide Phe-Gly-Gly-Phe of OFQ/N is similar to the sequence Tyr-Gly-Gly-Phe, which is present in all other opioid peptides. Despite the fact that N-terminal Tyr is essential for binding to the three known opioid receptors for dynorphins, endorphins, and enkephalins, OFQ/N binds to the NOP receptor, which belongs to the opioid receptor family and it was found that it does not bind opiates with high affinity [8]. The receptor was discovered in 1994 [9] and the NOP-OFQ/N system shows different pharmacological actions from the opioid receptor system, whereas different studies have outlined some roles of the NOP-OFQ/N system in nociception [10,11]. Although the route followed by OFQ/N in pain regulation is not perfectly clear, it is well recognized that this peptide is pain-inducing and causes hyperalgesia, as ceasing OFQ/N signaling was shown to raise pain threshold [12]. In any case, it is worth highlighting that the physiological activity of neuropeptides is regulated by the proteolytic processes that take place in the central nervous system. These enzymatic processes transform neuropeptides into shorter fragments, which can have distinct biological functions in comparison with the parent peptides from which they have been generated. In the case of OFQ/N, only very few studies investigate the metabolic processes undergone by the peptide and its fragments [13]. In this work, we report that insulin-degrading enzyme (IDE), an enzyme known for its multifaceted roles in cells [14,15,16,17] and its capability to degrade several different peptides [18,19,20,21], is capable of degrading OFQ/N in vitro, producing a characteristic proteolytic pattern. As IDE is present in the brain [22], which also contains Ins pools, it cannot be excluded that this enzyme indirectly participates in neural communication of pain signals and that neuropeptides involved in pain transmission may contribute to the regulation of IDE activity. Moreover, we have preliminary investigated the degradation of OFQ/N in the rat spinal cord, excluding the action of metalloproteases such as IDE by using EDTA, to screen for other enzymes that may also be responsible of the peptide proteolysis in vivo. Results show that, besides IDE, a cysteine protease is involved in the truncation of OFQ/N into shorter fragments, which remains to be identified.

## 2. Results

The IDE-mediated hydrolysis of OFQ/N in vitro yielded several peptides, which were detected and assigned by applying UPLC-HRMS. The high accuracy of the detected *m*/*z* values related to the most abundant peptides ions, coupled to their MS/MS acquisition has been used to undoubtedly identify the peptide sequence. The list of the most abundant hydrolytic peptides (Table 1) clearly shows that all the peptide bonds between Arg(8) and Lys(13) are cleaved by IDE, thus forming 1-8, 1-9, 1-10, 1-11, and 1-12 peptides. Only a N-truncated peptide (2-11) has been detected.

**Figure 1:**
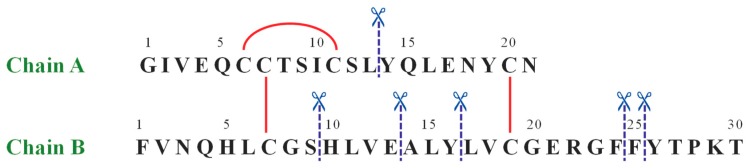
The amino acid sequence of human insulin. The red straight lines show intra- and inter-chain disulfide bridges, whereas the blue dashed lines label the IDE-catalyzed cleavage sites.

The same approach has been used to characterize by LC-MS the IDE-induced hydrolytic pattern of Ins. In Figure 1 the amino acid sequence of human Ins is reported.

**Figure 2:**
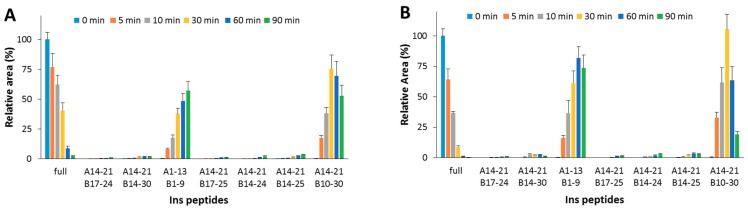
Hydrolytic pattern of the time-course Ins degradation catalyzed by IDE. The content of the full-length substrate (full) and the main hydrolytic peptides was detected in the absence (**A**) and in the presence (**B**) of OFQ/N, co-incubated with Ins and IDE at 37 °C for 90 min.

The detected peptides (Table 2 and Figure 2A) clearly confirm the attribution previously reported [23,24,25]. In the experimental conditions used, A1-13_B1-9 and A14-21_B10-30 are the main products. The content of the full-length substrate is more than halved after 30 min incubation and it almost disappears after 90 min. When OFQ/N was co-incubated with Ins and IDE (Figure 2B), the kinetic profiles of both the Ins degradation and the formation of the digested peptides did not significantly differ from those detected without OFQ/N.

**Figure 3:**
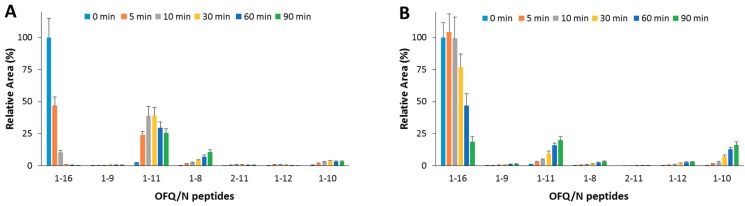
Hydrolytic pattern of the time-course OFQ/N degradation catalyzed by IDE. The content of the full-length substrate (1-16) and the main hydrolytic peptides was detected in the absence (**A**) and in the presence (**B**) of Ins, co-incubated with OFQ/N and IDE at 37 °C for 90 min.

As for the IDE-mediated OFQ/N degradation (Figure 3A), the substrate was almost completely degraded within 10 min; the first and main product (1-11) reached the highest amount after 30 min; 1-8 and 1-10 are also formed from the OFQ/N as well as from the 1-11 fragment processing.

When OFQ/N and Ins (1:1 molar ratio) were co-incubated with IDE (Figure 3B), OFQ/N ended up 80% of the original value after a 30 min reaction. The 1-11 fragment was still the main product, yet formation of this peptide was inhibited by the presence of Ins. Formation of 1-8 was reduced as well. On the contrary, the content of 1-10 fragment slightly increased.

**Figure 4:**
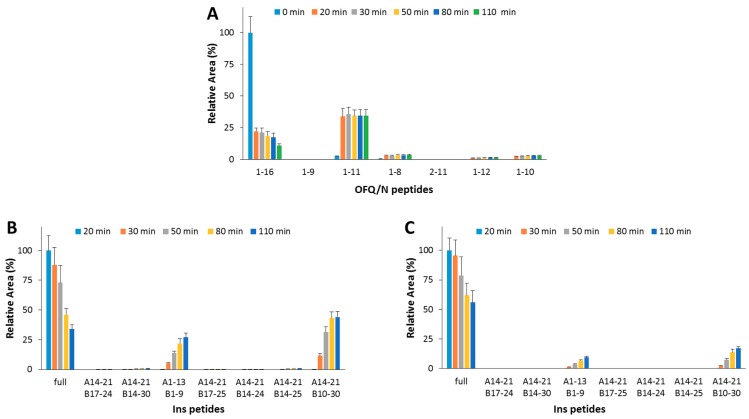
Hydrolytic pattern of the time-course OFQ/N (**A**) degradation catalyzed by IDE at 37 °C. OFQ/N was incubated with IDE for 20 min at 37 °C before the addition of Ins. The kinetic profile of the Ins degradation was analyzed when IDE was pre-incubated alone (**B**) or with OFQ/N (**C**) at 37 °C for 20 min.

In order to assess the effect (if any) of the OFQ/N hydrolytic fragments on the IDE-catalyzed Ins cleavage, OFQ/N was pre-incubated at 37 °C with IDE. After 20 min, Ins was added to the reaction mixture. At this point, the content of OFQ/N is 5-fold lower than its starting value (Figure 4A), whereas, after the addition of Ins, the amounts of neither the substrate (1-16) nor the main hydrolytic peptides (1-11, 1-10, and 1-8) significantly change. This clearly means that IDE-mediated digestion of the residual amount of OFQ/N after the pre-incubation step is inhibited by Ins even more than that reported for the co-incubation of the two substrates (Ins and OFQ/N). Focusing on the Ins digestion, the hydrolytic pattern was analyzed when IDE was pre-incubated alone or with OFQ/N for 20 min at 37 °C. In the first case (Figure 4B), the amount of full-length Ins gradually decreases and the content of released peptides increases. If OFQ/N is pre-incubated with IDE (Figure 4C), the hydrolysis and the formation rates of the Ins fragments significantly decrease. Differently from the results obtained for the co-incubation of Ins and OFQ/N with IDE, pre-incubation of OFQ/N with IDE clearly slows down the Ins hydrolysis.

**Figure 5:**
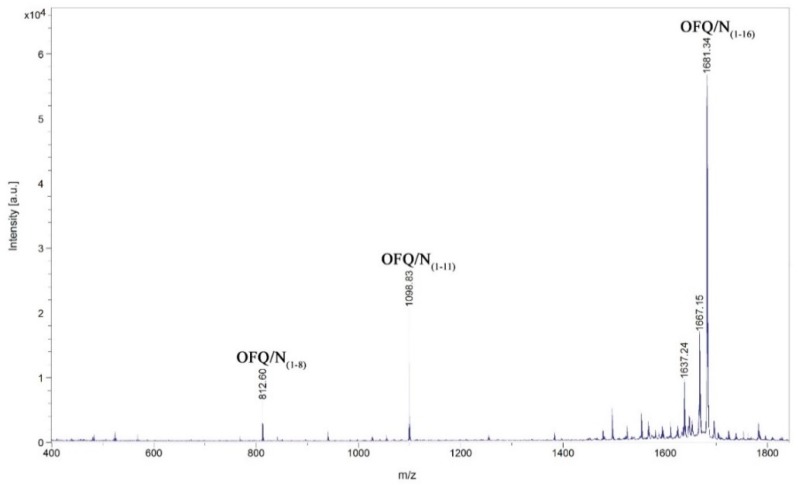
Identification by MALDI-MS of the major products after incubation of the neuropeptide OFQ/N with the homogenate obtained from the rat spinal cord. The incubation was performed using one microliter of the whole homogenate diluted with 19 µL of 20 mM Tris-HCl, 1 mM EDTA, pH 7.8, and 10 µL of Milli-Q water. The peptide concentration was 0.5 µg/mL, the incubation time was 30 min at 37 °C.

Taking into consideration the above reported results as well as the fact that IDE is present in CNS, degradation of OFQ/N in the rat spinal cord was then investigated and the same fragments found in vitro for the purified enzyme were detected. Afterwards, EDTA was added to the solution in order to verify if other proteases might also be involved in the degradation of OFQ/N in the rat spinal cord and the results are reported in Figure 5. The rationale of such study was to verify the hypothesis that processing of OFQ/N, at least partially, may depend on other proteases besides IDE. 

**Figure 6:**
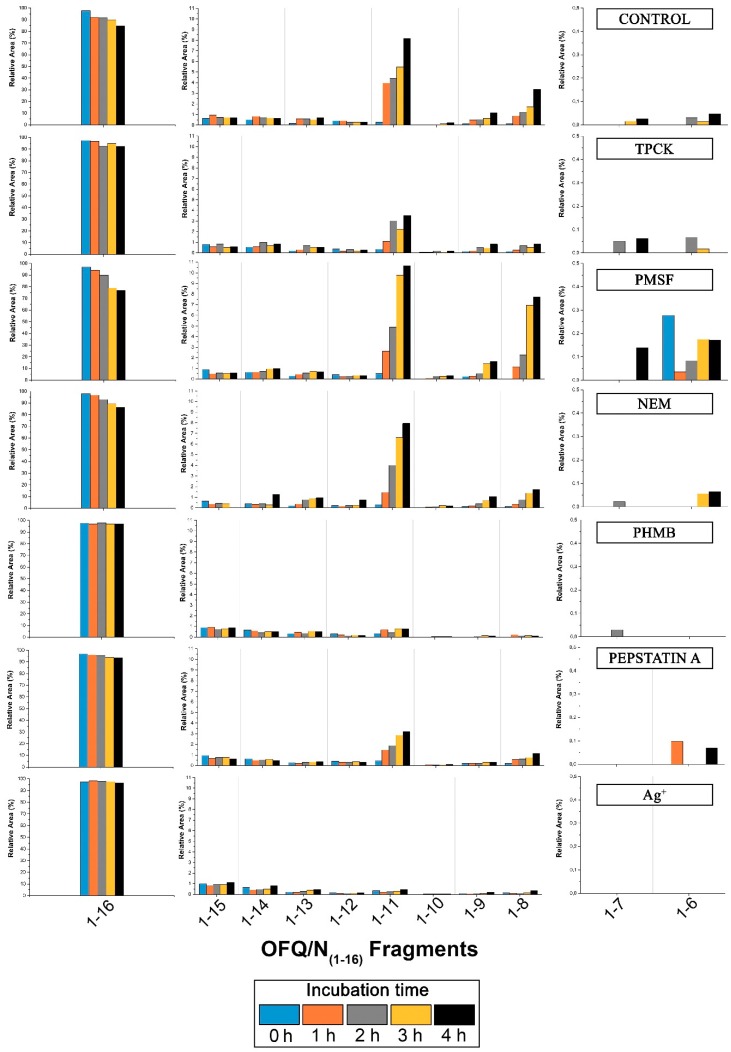
Time-course of the products after cleavage by rat spinal cord peptidases with the influence of different inhibitors on the metabolism. Bar graphs are relative to all the areas under the peaks detected by MALDI. On the left and on the right part of each histogram, relative areas of OFQ/N, fragments (1-7) and (1-6) are shown on a different scale due to the different values compared to all the other fragment areas.

Although in some cases the intensities are rather low, we could assign signals at 1098.83, 1496.19, 1383.08, 940.72, and 812.61 m/z, corresponding to the OFQ/N fragments (1-11), (1-14), (1-13), (1-9), and (1-8), respectively. The time course of the reaction in the presence of different classes of peptidases inhibitors is reported in Figure 6. In particular, TPCK and PMSF are serine proteases inhibitors; NEM and PHMB are cysteine proteases inhibitors; Pepstatin A is aspartyl proteases inhibitor; Ag^+^ is a strong metalloprotease inhibitor [18,19]. In each histogram presented in Figure 6, the presence of different fragments from (1-16), the parent peptide, is clearly visible. The first fragments (from 1-15 to 1-12) are probably produced by a carboxypeptidase, an enzyme able to cleave the initial peptide, one amino acid at a time, from the C-terminal side of the chain.

## 3. Discussion

We have found that Ins and OFQ/N compete for the interaction with IDE, thus affecting their own hydrolytic patterns. Indeed, the digestion of OFQ/N is significantly affected by the presence of Ins. On the contrary, the kinetic profile of the Ins hydrolysis does not clearly change in the presence of OFQ/N. However, the main hydrolytic peptides of OFQ/N exert an inhibitor activity towards the IDE-mediated Ins degradation and pre-incubation is necessary to compensate for the lower affinity of OFQ/N towards IDE if compared to the one between Ins and IDE.

The OFQ/N degradation in rat spinal cord has also been investigated and the most important findings concerns the (1-11), (1-7), and (1-6) fragments and their peak areas in the presence of different inhibitors, because these shorter sequences are the bioactive ones. Indeed, as reported in the literature, when intrathecally injected to mouse, (1-11) shows a naloxone-dependent analgesic action [26,27], whereas (1-7) fragment blocks hyperalgesia induced by OFQ/N injected in mice [28]. (1-11) fragment is later reduced to (1-6) fragment as a final product. The latter showed to have a bi-phasic effect in different analgesic tests: after ICV (Intracerebroventricular) and intrathecal injection it causes antinociception followed by a hyperalgesia. The cleavage of OFQ/N into the desired fragments is not efficiently inhibited by PMSF or TPCK-serine proteases inhibitors. The graph relative to the latter shows lower abundance of the (1-11) fragment compared to all the other histograms, but this could be due to the presence of methanol that stops enzyme activity in the incubation mixture. In fact, methanol was used to allow the dissolution of TPCK in water. Also, NEM does not show inhibition of the production of the desired bioactive fragments, whereas Pepstatin A slightly inhibits the formation of the (1-11) fragment. On the contrary, PHMB, a general cysteine proteases inhibitor, blocks the formation of the (1-11)fragment more than all the other inhibitors used during the experiment. This suggests that a cysteine protease is involved in the truncation of OFQ/N into shorter fragments with biological activity. Moreover, according to the data presented in Figure 6, PHMB inhibits the formation of (1-7) and (1-6) too.

## 4. Materials and Methods

IDE degradation assays: Insulin (Ins, 2 µM, purchased from Sigma, Milan, Italy) and nociceptin peptide (OFQ/N, 2 µM, synthesized by the Fmoc solid phase synthesis and purified in our laboratory using RP HPLC) were tested for their purity using electrospray mass spectrometry (over 98% purity), and hydrolyzed in the presence of insulin-degrading enzyme (IDE, 30 nM, purchased from Giotto Biotech, Florence, Italy) in phosphate buffer 1 mM (pH 7.4, Sigma). We applied 1-16 sequence of OFQ/N because there is no difference in binding of this fragment to NOP receptors. Ins and OFQ/N were hydrolyzed both separately and in a mixture. Moreover, a pre-incubation of OFQ/N with IDE for 20 min was also performed before the addition of Ins. In order to quench the reaction, the samples were diluted 1:1 with 1% TFA aqueous solution.

The peptide content of all samples was analyzed using a Q Exactive hybrid quadrupole-Orbitrap mass spectrometer (Thermo Scientific, Milan, Italy) coupled to an Ultimate 3000 HPLC RSLCnano system (Dionex Thermo Scientific, Milan, Italy) through an EASY-Spray source (Thermo Scientific, Milan, Italy) by using the instrument settings previously reported [29].

Tissue homogenate preparation: One rat spinal cord tissue was homogenized in 1 mL of ice-cold 20 mM Tris-HCl (Sigma, Milan, Italy) and 1 mM EDTA (Sigma, Milan, Sigma) using mechanical homogenizer. The obtained homogenate was centrifuged at 5000× *g* for 15 min at 4 °C. The supernatant was then centrifuged at 20,000× *g* for 15 min at 4 °C. Finally, the supernatant was divided into small aliquots and kept frozen at −80 °C [13].

Tissue extract (supernatant) incubation assays: Incubations were performed using one microliter of tissue extract diluted with 19 µL of 20 mM Tris-HCl 1 mM EDTA buffer pH 7.8 and 10 µL of Milli-Q water. Then, the reaction was initiated by the addition of 10 µL of OFQ/N 0.3 mM and continued at 37 °C for 30 min. Then, 40 µL of ice-cold methanol was used to stop the reaction. Incubation was repeated as mentioned above but adding different peptidase inhibitors. The inhibitors used for the incubation were: TPCK (tosyl phenylalanyl chloromethyl ketone) 1 mM, PMSF (phenylmethylsulfonyl fluoride) 1 mM, NEM (N-ethylmaleimide) 1 mM, PHMB (4-(hydroxymercuri)benzoic acid) 0.25 mM, pepstatin A 0.01 mM, and Ag^+^ 1 mM. Enzyme assay including spinal cord homogenate was also validated using dynorphin B, as described previously [30]. One microliter aliquots taken from each incubation mixture were analyzed using MALDI mass spectrometry. α-Cyano-4-hydroxycinnamic acid (4-HCCA) was used as matrix, and the final samples were spotted onto AnchorChip™ plate (Bruker Daltonics, 2013). The standard used for the calibration of the instrument is the Peptide Calibration Standard II by Bruker Daltonics, Macerata, Italy. Details and parameters of the instrument set-up were: mass range: 340–4000 Da; detector gain/reflector: 6.3x (2480 V); sample rate and digitizer settings: 2.50 GS/s; smartbeam parameter set: 4_large; positive ion mode; matrix suppression: off; random walk: complete sample; shots at raster spot: 200; limit diameter to: 800 µm; laser power: 20%; shots: 5000.

## 5. Conclusions

We have investigated the degradation of the OFQ/N peptide in vitro by purified IDE. Additionally, we performed a preliminary screen for other peptidases potentially cleaving OFQ/N in the rat spinal cord. We have found that IDE is capable of degrading the neuropeptide to generate shorter fragments that are reported to exhibit an anti-nociceptive effect. We have shown that, in vitro, Ins slows down the degradation of the OFQ/N peptide by IDE, whereas the OFQ/N peptide does not affect Ins degradation by IDE. However, the OFQ/N smaller fragments produced by the action of IDE on the OFQ/N peptide affects Ins proteolysis by IDE, as demonstrated by pre-incubating the enzyme with the OFQ/N peptide before Ins addition. These results demonstrate that Ins dyshomeostasis due to the type 2 or type 1 diabetes can have a direct impact on pain transmission and pain threshold through an IDE mediated mechanism. This result is in line with all the recent findings, which demonstrate a multifaceted role for this enzyme in the brain [14,15,16,31].

Moreover, preliminary results with the use of rat spinal cord indicate that a cysteine protease, besides IDE, could be involved in the truncation of OFQ/N into shorter fragments in vitro.

Future experiments, carried out in the presence of specific IDE inhibitors and Ins fragments, will allow assessment of the relevance of our findings in vivo, thus elucidating whether nociceptin degradation by IDE is the main process responsible for the reported higher pain threshold in diabetic patients or if other biochemical pathways are also involved [32].

## Figures and Tables

**Table 1:** List of all the identified IDE-promoted OFQ/N hydrolytic peptides and their LC-MS features

Abbreviation	Amino acid sequence	Measured *m*/*z*	Calculated *m*/*z*	*z*	MW	Δm (*ppm*)	RT (*min*)
1-16	FGGFTGARKSARKLAN	420.9884	420.9878	4	1682.944	1.4	16.0
1-9	FGGFTGARK	470.7546	470.7536	2	942.5145	2.1	16.5
1-11	FGGFTGARKSA	366.8609	366.8612	3	1100.584	−0.8	16.6
1-8	FGGFTGAR	406.7068	406.7061	2	814.4195	1.7	18.0
2-11	GGFTGARKSA	476.2534	476.2540	2	953.5152	−1.3	16.6
1-12	FGGFTGARKSAR	418.8953	418.8949	3	1256.685	1.0	15.8
1-10	FGGFTGARKS	343.1827	343.1822	3	1029.547	1.5	16.4

**Table 2:** List of all the identified IDE-promoted Ins hydrolytic peptides and their LC-MS features. The sulfur atoms involved in disulphide bonds are reported in bold and underlined (*Amino acid sequence* column). “A” denotes insulin chain A and “B” denotes insulin chain B.

Abbr.	Amino acid sequence	Meas. *m/z*	Calc. *m/z*	*z*	MW	Δm (*ppm*)	RT (*min*)
A1-21 B1-30	GIVEQ**CC**TSI**C**SLYQLENY**C**N FVNQHL**C**GSHLVEALYLV**C**GERGFFYTPKT	1162.3356	1162.3335	5	5810.690	1.8	25.0
A14-21 B17-24	YQLENY**C**N LV**C**GERGF	641.9489	641.9503	3	1926.858	−2.2	19.8
A14-21 B14-30	YQLENY**C**N ALYLV**C**GERGFFYTPKT	752.8557	752.8544	4	3011.418	1.7	23.0
A1-13 B1-9	GIVEQC**C**TSICSL FVNQHL**C**GS	785.6805	785.6794	3	2358.045	1.4	21.7
A14-21 B17-25	YQLENY**C**N LV**C**GERGFF	690.9723	690.9731	3	2073.927	−1.2	21.9
A14-21 B14-24	YQLENY**C**N ALYLV**C**GERGF	757.6795	757.6785	3	2274.043	1.3	22.4
A14-21 B14-25	YQLENY**C**N ALYLV**C**GERGFF	806.7005	806.7013	3	2421.111	−1.0	23.4
A14-21 B10-30	YQLENY**C**N HLVEALYLV**C**GERGFFYTPKT	872.4189	872.4179	4	3489.672	1.1	24.1

## References

[1] Kaul K., Tarr J.M., Ahmad S.I., Kohner E.M., Chibber R. (2012). Introduction to diabetes mellitus. Adv. Exp. Med. Biol..

[2] Saidian M., Lakey J.R.T., Ponticorvo A., Rowland R., Baldado M., Williams J., Pronda M., Alexander M., Flores A., Shiri L. (2019). Characterisation of impaired wound healing in a preclinical model of induced diabetes using wide-field imaging and conventional immunohistochemistry assays. Int. Wound J..

[3] Kaur P., Sharma A.K., Nag D., Das A., Datta S., Ganguli A., Goel V., Rajput S., Chakrabarti G., Basu B. (2019). Novel nano-insulin formulation modulates cytokine secretion and remodeling to accelerate diabetic wound healing. Nanomedicine.

[4] Masood N., Ahmed R., Tariq M., Ahmed Z., Masoud M.S., Ali I., Asghar R., Andleeb A., Hasan A. (2019). Silver nanoparticle impregnated chitosan-PEG hydrogel enhances wound healing in diabetes induced rabbits. Int. J. Pharm..

[5] Telli O., Cavlak U. (2006). Measuring the pain threshold and tolerance using electrical stimulation in patients with Type II diabetes mellitus. J. Diabetes Complicat..

[6] Kukidome D., Nishikawa T., Sato M., Igata M., Kawashima J., Shimoda S., Matsui K., Obayashi K., Ando Y., Araki E. (2016). Measurement of small fibre pain threshold values for the early detection of diabetic polyneuropathy. Diabet. Med..

[7] Takeshita N., Yamaguchi I. (1997). Insulin attenuates formalin-induced nociceptive response in mice through a mechanism that is deranged by diabetes mellitus. J. Pharmacol. Exp. Ther..

[8] Mollereau C., Parmentier M., Mailleux P., Butour J.L., Moisand C., Chalon P., Caput D., Vassart G., Meunier J.C. (1994). ORL1, a novel member of the opioid receptor family. Cloning, functional expression and localization. FEBS Lett..

[9] Bunzow J.R., Saez C., Mortrud M., Bouvier C., Williams J.T., Low M., Grandy D.K. (1994). Molecular cloning and tissue distribution of a putative member of the rat opioid receptor gene family that is not a mu, delta or kappa opioid receptor type. FEBS Lett..

[10] Calo’ G., Guerrini R., Rizzi A., Salvadori S., Regoli D. (2000). Pharmacology of nociceptin and its receptor: a novel therapeutic target. Br. J. Pharmacol..

[11] Calo’ G., Rizzi A., Bigoni R., Guerrini R., Salvadori S., Regoli D. (2002). Pharmacological profile of Nociceptin/Orphanin FQ receptors. Clin. Exp. Pharmacol. Physiol..

[12] Meunier J.C., Mollereau C., Toll L., Suaudeau C., Moisand C., Alvinerie P., Butour J.L., Guillemot J.C., Ferrara P., Monsarrat B. (1995). Isolation and structure of the endogenous agonist of opioid receptor-like ORL1 receptor. Nature.

[13] Suder P., Kotlinska J., Smoluch M.T., Sällberg M., Silberring J. (1999). Metabolic fate of nociceptin/orphanin FQ in the rat spinal cord and biological activity of its released fragment. Peptides.

[14] Grasso G., Lanza V., Malgieri G., Fattorusso R., Pietropaolo A., Rizzarelli E., Milardi D. (2015). The insulin degrading enzyme activates ubiquitin and promotes the formation of K48 and K63 diubiquitin. Chem. Commun. (Camb).

[15] Sbardella D., Tundo G.R., Coletta A., Marcoux J., Koufogeorgou E.I., Ciaccio C., Santoro A.M., Milardi D., Grasso G., Cozza P. (2018). The insulin-degrading enzyme is an allosteric modulator of the 20S proteasome and a potential competitor of the 19S. Cell Mol. Life Sci..

[16] Bellia F., Lanza V., Ahmed I.M.M., Garcia-Vinuales S., Veiss E., Arizzi M., Calcagno D., Milardi D., Grasso G. (2019). Site directed mutagenesis of insulin-degrading enzyme allows singling out the molecular basis of peptidase versus E1-like activity: the role of metal ions. Metallomics.

[17] Tundo G.R., Sbardella D., Ciaccio C., Grasso G., Gioia M., Coletta A., Polticelli F., Di Pierro D., Milardi D., Van Endert P. (2017). Multiple functions of insulin-degrading enzyme: a metabolic crosslight?. Crit. Rev. Biochem. Mol. Biol..

[18] Grasso G., Pietropaolo A., Spoto G., Pappalardo G., Tundo G.R., Ciaccio C., Coletta M., Rizzarelli E. (2011). Copper(I) and Copper(II) Inhibit Aβ Peptides Proteolysis by Insulin-Degrading Enzyme Differently: Implications for Metallostasis Alteration in Alzheimer’s Disease. Chem. Eur. J..

[19] Grasso G., Salomone F., Tundo G.R., Pappalardo G., Ciaccio C., Spoto G., Pietropaolo A., Coletta M., Rizzarelli E. (2012). Metal ions affect insulin-degrading enzyme activity. J. Inorg. Biochem..

[20] Bellia F., Grasso G. (2014). The role of copper(II) and zinc(II) in the degradation of human and murine IAPP by insulin-degrading enzyme. J. Mass. Spectrom..

[21] Ciaccio C., Tundo G.R., Grasso G., Spoto G., Marasco D., Ruvo M., Gioia M., Rizzarelli E., Coletta M. (2009). Somatostatin: a novel substrate and a modulator of insulin-degrading enzyme activity. J. Mol. Biol..

[22] Gray S.M., Barrett E.J. (2018). Insulin transport into the brain. Am. J. Physiol. Cell Physiol..

[23] Grasso G., Rizzarelli E., Spoto G. (2007). AP/MALDI-MS complete characterization of the proteolytic fragments produced by the interaction of insulin degrading enzyme with bovine insulin. J. Mass. Spectrom..

[24] Grasso G., Rizzarelli E., Spoto G. (2009). The proteolytic activity of insulin-degrading enzyme: a mass spectrometry study. J. Mass. Spectrom..

[25] Bellia F., Pietropaolo A., Grasso G. (2013). Formation of insulin fragments by insulin-degrading enzyme: the role of zinc(II) and cystine bridges. J. Mass. Spectrom..

[26] Rossi G.C., Leventhal L., Bolan E., Pasternak G.W. (1997). Pharmacological characterization of orphanin FQ/nociceptin and its fragments. J. Pharmacol. Exp. Ther..

[27] Rossi G.C., Perlmutter M., Leventhal L., Talatti A., Pasternak G.W. (1998). Orphanin FQ/nociceptin analgesia in the rat. Brain Res..

[28] Sakurada C., Sakurada S., Katsuyama S., Sasaki J., Tan-No K., Sakurada T. (1999). Involvement of tachykinin NK1 receptors in nociceptin-induced hyperalgesia in mice. Brain Res..

[29] Bellia F., Lanza V., Garcia-Vinuales S., Ahmed I.M.M., Pietropaolo A., Iacobucci C., Malgieri G., D’Abrosca G., Fattorusso R., Nicoletti V.G. (2019). Ubiquitin binds the amyloid β peptide and interferes with its clearance pathways. Chem. Sci..

[30] Silberring J., Nyberg F. (1989). A novel bovine spinal cord endoprotease with high specificity for dynorphin B. J. Biol. Chem..

[31] Grasso G., Mielczarek P., Niedziolka M., Silberring J. (2014). Metabolism of Cryptic Peptides Derived from Neuropeptide FF Precursors: The Involvement of Insulin-Degrading Enzyme. Int. J. Mol. Sci..

[32] Madiraju S.R.M., Poitout V. (2007). GPCRs and Insulin Secretion: 119 and Counting. Endocrinology.

